# La fistule pancréatico-pleurale chez l’enfant: étiologie rare d’une pleurésie de grande abondance

**DOI:** 10.11604/pamj.2017.26.240.9003

**Published:** 2017-04-26

**Authors:** Aida Daib, Youssef Hellal, Malek Boughdir, Rabiaa Ben Abdallah, Nejib Kaabar

**Affiliations:** 1 Département de Chirurgie Pédiatrique, Hôpital Habib Thameur, 1008 Tunis, Tunisia

**Keywords:** Faux kyste du pancréas, fistule pleurale, pleurésie, enfant, False cyst of the pancreas, pleural fistula, pleurisy, child

## Abstract

La fistule pancréatico-pleurale est une complication très rare des faux kystes du pancréas. L’objectif de notre article est de décrire cette pathologie rare chez l’enfant. On insistera sur l’importance de l’évoquer devant une pleurésie de grande abondance même en l’absence des signes digestifs. Nous décrivons l’observation d’un enfant âgé de 2 ans ayant un faux kyste du pancréas compliqué d’une fistule pleurale découvert suite à une pleurésie de grande abondance sans signes digestifs associés.

## Abstract (en)

Pancreatic-pleural fistula is a very rare complication of false cysts of the pancreas. Our study aimed to describe this rare pathology affecting children. We emphasized the importance of evoking this diagnosis in the presence of great abundant pleurisy even in the absence of the digestive signs. We here report the case of a 2-year old child with false cyst of the pancreas complicated by pleural fistula detected after great abundant pleurisy with no digestive signs associated.

## Introduction

La fistule pancréatico-pleurale est une complication rare des faux kystes du pancréas. Elle est secondaire à des perturbations au niveau des canaux pancréatiques, donnant ainsi l’accès aux enzymes pancréatiques à la cavité pleurale. La symptomatologie abdominale peut manquer dans certains cas. Nous rapportons un cas de fistule pancréatico-pleurale chez un garçon de 2 ans découverte par une pleurésie de grande abondance.

## Patient et observation

**Figure 1:**
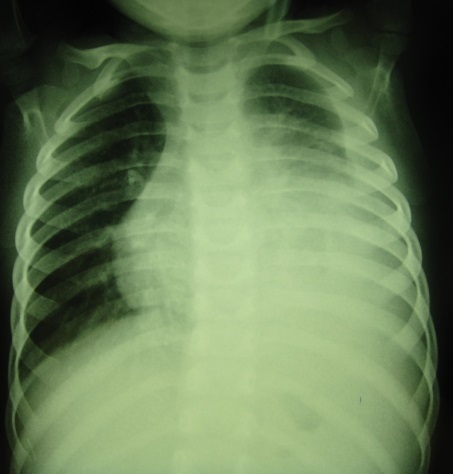
Radiographie de thorax montrant une pleurésie de grande abundance

Il s’agissait d’un enfant de sexe masculin, âgé de 2 ans, ayant présenté depuis un mois des douleurs abdominales et des vomissements négligés par les parents et qui étaient de résolution spontanée. Il a été hospitalisé pour épanchement pleural gauche découvert suite à une toux persistante. A l’examen, on notait un syndrome d’épanchement pleural liquidien gauche sans troubles respiratoires associés. Biologiquement, il n’y avait pas d’anomalies. La radiographie thoracique avait montré un épanchement pleural gauche de grande abondance avec refoulement des éléments du médiastin (Figure 1). A la ponction, le liquide était hématique de type exsudatif. Un drain était posé en urgence et on notait une nette régression de l’épanchement à la radiographie de contrôle. Dix jours après l’ablation du drainage, il a présenté une récidive de la pleurésie.

**Figure 2:**
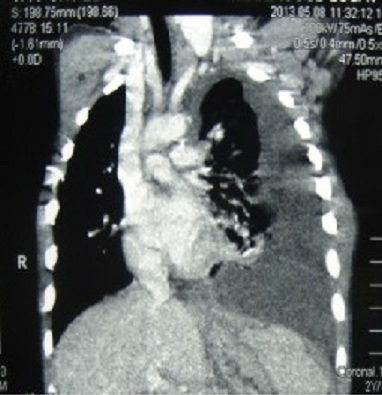
Collection thoracique à la tomodensitométrie

**Figure 3:**
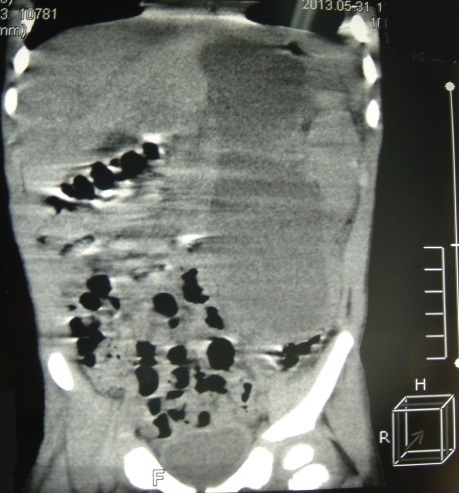
Extension intra-abdominale de la collection thoracique

Une tomodensitométrie thoraco-abdominale était faite, elle a conclu à une volumineuse collection médiastinale postérieure de contenu mixte aéro-liquidien étendue à l’abdomen à travers l’orifice hiatale se continuant par une deuxième collection de même caractéristique rétro-gastrique (Figure 2, Figure 3). A l’échographie abdominale, on notait la présence d’une collection liquidienne interspléno-gastrique de 3 centimètres de grand axe sans autres anomalies associées. Le diagnostic d’une duplication gastrique de localisation thoraco-abdominale était évoquer d’où le patient était opéré.

Il avait eu une laparotomie médiane avec découverte en per opératoire d’une masse liquidienne intimement accolée à la tête et au corps du pancréas, étendue au thorax à travers l’orifice hiatal, dont la paroi ne comportait pas de couche musculaire. La ponction ramenait 250 millilitres de liquide brunâtre qu’on adressait pour examen biochimique. La lipase était supérieure à 80000UI/ml et l’amylase supérieure à 20000UI/ml. Le diagnostic d’un faux kyste du pancréas fistulisé dans la plèvre était retenu. On faisait une résection du dôme saillant avec curetage de la coque et un drainage par un drain de Redon. L’examen anatomopathologique avait confirmé le diagnostic : paroi kystique remaniée sans structures tissulaires reconnaissables.

En postopératoire, le patient était mis sous sondostatine et une antibiothérapie, avec des suites simples : la radiographie thoracique était satisfaisante avec à l’échographie abdominale absence d’épanchement ou collection intra péritonéale. Les suites étaient simples avec un recul de 2 ans.

## Discussion

La fistule pancréatico-pleurale est une entité rare avec une fréquence inférieure à 1% [1-4]. Elle survient généralement chez les malades porteurs d’une pancréatite chronique (alcoolique). La fistule diaphragmatique permet la communication entre les canaux pancréatiques et la cavité pleurale. Chez les enfants, seulement une dizaine de cas sont rapportées dans la littérature [5] et sont tous en rapport avec un faux kyste du pancréas. L’étiologie de la pancréatite est rarement identifiable. Deux cas dans la littérature ont été associés à une malformation congénitale des canaux pancréatique [6]. Dans notre observation, la cause de la pancréatite est restée inconnue.

La symptomatologie respiratoire est généralement au premier plan faite d’une pleurésie de grande abondance avec ou sans troubles respiratoires associés [4, 5]. La ponction du liquide pleural ramène le plus souvent un liquide séro-hématique [5]. Son étude biochimique montre un taux élevé d’amylase ce qui oriente le diagnostic. La symptomatologie abdominale est rarement présente [4, 5]. Notre malade s’est présenté pour une symptomatologie respiratoire mais avec une notion des douleurs abdominales datant d’un mois et négligées par les parents. Le liquide pleural a été séro-hématique mais la recherche d’amylase dans ce liquide n’a été pas pratiquée.

Un scanner thoraco-abdominal permet de poser le diagnostic en montrant le faux kyste du pancréas et le trajet fistuleux qui a été l’orifice hiatal chez notre malade et à travers le hiatus aortique chez Duncan et al [5]. Dans notre série, la présence d’une composante aérienne au niveau de l’épanchement pleural a détourné le diagnostic vers une duplication gastrique. Le diagnostic de fistule pancréatico-pleurale a été porté en per-opératoire. Nous avons rapporté cette composante aérienne au drainage. La cholangiopancréatographie retrograde endoscopique est un excellent examen permettant d’illustrer l’anatomie aussi bien des canaux pancréatiques et de la fistule pleurale [5, 7].

Certaines équipes préfèrent de commencer par un traitement conservateur associant un drainage thoracique, un traitement médical à base de somatostatine et une alimentation parentérale [4, 5]. Cette démarche thérapeutique a un taux de succès qui avoisine 50% des malades adultes mais avec un haut risque de récidive [1, 5]. Des autres auteurs supportent l’attitude chirurgicale qui consiste en une résection pancréatique distale avec fermeture de la fistule pleurale. Une pancréatico-jéjunostomie peut être nécessaire en cas de pathologie pancréatique proximale [4, 5]. Dans notre cas, le traitement a été d’emblé chirurgical vu que le diagnostic a été méconnu en préopératoire. Le traitement chirurgical a consisté en une simple résection du dome saillant du faux kyste sans recours à une pancréatectomie même partielle.

## Conclusion

La fistule pancréatico-pleurale est une complication rare des faux kystes du pancréas chez l’enfant. Le diagnostic doit être évoqué devant une pleurésie dans un contexte d’apyrexie même en l’absence des signes digestifs. Une simple ponction du liquide pleural avec recherche du taux d’amylase peut orienter le diagnostic. La confirmation est radiologique. Un traitement conservateur peut être tenté initialement. Le traitement chirurgical à minima peut donner des bons résultats sans recours à une intervention lourde pour l’enfant.
